# Detecting undiagnosed dementia using medicare claims data

**DOI:** 10.1002/alz70856_107041

**Published:** 2026-01-09

**Authors:** MacKenzie Tweardy, Keith J Yoder, Spencer Gerrol, Ché Lucero

**Affiliations:** ^1^ SPARK Neuro, Inc., New York, NY, USA

## Abstract

**Background:**

The Center for Medicare and Medicaid Services advocates for cognitive assessments during annual wellness visits. However, traditional screening tools such as the Mini‐Mental State Exam require 15 to 20 minutes to administer and interpret, exceeding the average time of an entire primary care visit. With approximately 60 million elderly Americans, manual screening is infeasible. Rather than relying on manually administered tests, a more practical approach may be to leverage existing healthcare data. Given that nearly all healthcare interactions generate insurance claims, claims data may provide a scalable and widely accessible alternative for identifying dementia cases.

**Method:**

We analyzed 40 million Medicare claims across 1.9 million individuals over a five year period (2018 ‐ 2022) to develop a model to predict undiagnosed dementia. We took all beneficiaries who had a dementia code (e.g. F00 – Dementia in Alzheimer's disease) in their record as the positive cases, and the first appearance in their record as the incident diagnosis. It takes an average of 4.9 months from initial cognitive complaint to dementia diagnosis. Therefore, we excluded all claims data in the 6 months leading up to diagnosis for the positive cases to ensure we were not taking advantage of claims generated during the diagnostic process. To create our feature set, we extracted literature‐based dementia risk factors and comorbidities and broke each beneficiary's claims into separate time periods. Within each period, we counted the number of times each code appeared, and then normalized the counts by the length of the time period. Finally, we trained a gradient‐boosted decision tree (XGBoost) to identify whether a set of claims data indicated the beneficiary was “positive” or “negative” for dementia. We evaluated performance using cross‐validation.

**Result:**

The model achieved an accuracy of 93.9%, with 44.3% sensitivity and 98.8% specificity.

**Conclusion:**

Intelligent interpretation of claims data can identify undocumented cases of dementia. Application within a health system could allow physicians to fruitfully focus their attention and spend the time on patients who need it.

